# Expert Consensus on Clinical Decision Making in the Disease Trajectory of Oropharyngeal Dysphagia in Adults: An International Delphi Study

**DOI:** 10.3390/jcm12206572

**Published:** 2023-10-17

**Authors:** Renée Speyer, Mathieu Balaguer, Emmanuelle Cugy, Clémence Devoucoux, Sylvain Morinière, Gaëlle Soriano, Eric Vérin, Virginie Woisard

**Affiliations:** 1 Department Special Needs Education, University of Oslo, 0318 Oslo, Norway; 2 Curtin School of Allied Health, Faculty of Health Sciences, Curtin University, Perth 6102, Australia; 3 Department of Otorhinolaryngology and Head and Neck Surgery, Leiden University Medical Centre, 2333 ZA Leiden, The Netherlands; 4 Institut de Recherche en Informatique de Toulouse, Centre national de la Recherche Scientifique, University of Toulouse, 31062 Toulouse, France; mathieu.balaguer@irit.fr; 5 Department of Medicine, Maieutic and Allied Health, Faculty of Health, University of Toulouse, Paul Sabatier Toulouse III, 133 Route de Narbonne, 31062 Toulouse, France; devoucoux.c@chu-toulouse.fr (C.D.); woisard.virginie@iuct-oncopole.fr (V.W.); 6 Department of Physical and Rehabilitation Medicine, Arcachon Hospital, 33260 La Teste de Buch, France; emmanuelle.cugy@chu-bordeaux.fr; 7 Department of Physical and Rehabilitation Medicine, Tastet Girard Hospital, CEDEX, 33076 Bordeaux, France; 8 Department of Otorhinolaryngology and Head and Neck Surgery, Larrey Hospital, University Hospital of Toulouse, 31400 Toulouse, France; 9 Department of Otorhinolaryngology and Head and Neck Surgery, Tours University Hospital, 37000 Tours, France; sylvain.moriniere@univ-tours.fr; 10 Otorhinolarygology Department, University François Rabelais of Tours, 10 Bd Tonnellé, 37032 Tours, France; 11 Department of Geriatrics, Toulouse University Hospital, 31000 Toulouse, France; soriano.g@chu-toulouse.fr; 12 GRHVN UR 3830, Research Group on Ventilatory and Neurological Handicap, Laboratoire de Chirurgie Experimentale, University of Rouen Normandie, 76000 Rouen, France; eric.verin@chu-rouen.fr; 13 Department of Pulmonary Rehabilitation, CHU Rouen, UR 3830, Normandie University, UNIROUEN, 76000 Rouen, France; 14 Neuro-Psycho-Linguistic Lab UR 4156, University of Toulouse, Jean Jaurès Toulouse II, 5, Allée Antonio Machado, CEDEX 9, 31058 Toulouse, France; 15 Department of Supportive Care, Cancer University Institute of Toulouse Oncopole, 1 avenue Irène Joliot-Curie, CEDEX 9, 31059 Toulouse, France

**Keywords:** deglutition, swallowing disorders, survey, questionnaire, framework, prognosis, risk, impairment, severity

## Abstract

To date, no consensus exists on the complex clinical decision-making processes involved in oropharyngeal dysphagia, or swallowing disorders. This study aimed to develop an international consensus on a clinical decision tree for the disease trajectory of oropharyngeal dysphagia in adults, taking into account physiological impairments of swallowing, risk factors for the development of complications from oropharyngeal dysphagia, and prognostic factors for treatment outcomes. Using the Delphi technique, consensus was achieved among dysphagia experts across 31 countries, resulting in a total of 10 physiological impairments, 23 risk factors and 21 prognostic factors identified as relevant factors in the clinical decision-making process. Factors most contributing to the severity of oropharyngeal dysphagia were ‘Aspiration’, ‘Incomplete ejection or failure to eject aspirated materials from the airways’, ‘Weak or absent cough’, ‘Choking’ and ‘Sensory deficits in the oropharynx’. To connect the existing theoretical framework to clinical practice, future research will develop the current findings by corroborating the domains based on relevant factors for clinical decision making and those that contribute to the severity of oropharyngeal dysphagia.

## 1. Introduction

Dysphagia (i.e., swallowing problems) is a symptom or a collection of symptoms of underlying anatomical abnormalities, or impairments and disorders in cognitive, sensory and motor acts involved in the transport of, mainly, food and drink from the oral cavity towards the stomach. Dysphagia may lead to reduced efficiency and safety of swallowing, failure to maintain hydration and nutrition, risk of choking and aspiration, leading to pulmonary complications and reduced quality of life [1]. In contrast to esophageal dysphagia, which typically results from a motility disorder or obstruction in the esophagus, oropharyngeal dysphagia refers to abnormalities affecting the upper esophageal sphincter, pharynx, larynx, and/or tongue.

The prevalence of oropharyngeal dysphagia in the general population has been estimated to range between 2.3 and 16% [2], but in selected patient populations pooled prevalence has been reported to be as high as 42% in stroke [3], 50.9% in cerebral palsy [4] and 72.4% in dementia [5]. Prevalence numbers may depend on the severity of underlying diseases, screening or assessment tools used to identify dysphagia, and healthcare settings [6]. To date, no international consensus exists on which outcome measures to use in order to identify and monitor oropharyngeal dysphagia [7,8]. Moreover, there is no agreement on how clinical decisions are made regarding the management and care of patients with oropharyngeal dysphagia.

Few publications are available on the subject of decision making for dysphagia relevant for selected patient populations, for instance head and neck oncology [9] and stroke [10]. However, these studies are based on limited literature and restricted numbers of clinical cases without presenting a complete overview or framework based on international consensus between healthcare professionals involved in the disease trajectory of oropharyngeal dysphagia. Alternatively, a more generic approach can be taken by applying the four-topic framework developed by Jonsen et al. [11] to support ethical reasoning in clinics. The framework is used through the identification of four domains and principles: medical indications referring to diagnostic and therapeutic interventions (i.e., the principle of beneficence and nonmaleficence); patient preferences in treatment (i.e., the principle of respect for autonomy); patient quality of life prior to and following treatment (i.e., the principle of beneficence and nonmaleficence and respect for autonomy); and, contextual features, for example, social, institutional, financial and legal settings influencing medical decisions (i.e., the principle of justice and fairness). Although this approach frames ethical dilemmas and can be applied in clinics, the framework only organises dilemmas, and clinicians are still required to use their own judgment in examining the respective weights of ethical principles [12].

The process of clinical decision making for oropharyngeal dysphagia in adults involves the identification of three factors across the disease trajectory: (1) physiological impairments of swallowing (or swallowing disability) resulting in inefficient and/or unsafe swallowing; (2) risk factors for the development of complications from oropharyngeal dysphagia; and, (3) prognostic factors for treatment outcome. Swallowing efficiency refers to the ability to transfer a bolus, secretions and/or any other material from the oral cavity, nasal cavity and paranasal sinuses, tracheobronchial tree or esophagus to the stomach without post-swallow residue, whereas swallowing safety is the ability to transfer material to the stomach without penetration and/or aspiration into the lower airways [13]. Risk factors for the development of complications from oropharyngeal dysphagia (e.g., pulmonary complications or aspiration pneumonia, reduced nutrition or oral intake, and/or poor health-related quality of life or depression) refer to patient characteristics related to health status or comorbidities, and/or health consequences due to the physiological impairment of swallowing. For example, health consequences due to tracheal residue (physiological impairment) may lead to the development of aspiration pneumonia (complication) in the presence of pulmonary disease, e.g., COPD (risk factor). Prognostic factors may impact the success of any treatment for oropharyngeal dysphagia and/or treatment of underlying etiology.

The aim of this study is to develop an international consensus on a clinical decision-making tree for the disease trajectory of oropharyngeal dysphagia in adults. To achieve this, the Delphi technique will be utilised, with consideration given to the physiological impairments of swallowing, risk factors for the development of complications from oropharyngeal dysphagia, and prognostic factors for treatment outcome.

## 2. Methods

### 2.1. Study Design

This study used the Delphi technique to develop international consensus on a framework for clinical decision making for the disease trajectory of oropharyngeal dysphagia in adults, taking into account: (1) *physiological impairments* of swallowing resulting in efficient and/or unsafe swallowing; (2) *risk factors* for the development of complications from oropharyngeal dysphagia (referring to patient characteristics in relation to health status or comorbidities, and/or health consequences due to physiological impairment of swallowing); and, (3) *prognostic factors* for treatment outcome and/or treatment of underlying etiology.

Group consensus between experts was achieved using a series of Delphi rounds as part of a structured process [14] during which consecutive online surveys (e-Delphi) were modified based on participants’ percentage of agreement and feedback from preceding survey rounds. Delphi rounds continued until group consensus was achieved or it became obvious that consensus would not be reached. To avoid bias, participants remained anonymous throughout all Delphi rounds. For all rounds, the same experts were invited although some participants may have chosen to withdraw during the Delphi process.

### 2.2. Participants

Participants were eligible to participate in the Delphi study if they: (1) were fluent in English (i.e., able to use English adequately for work and study purposes); and, (2) had spent five years full-time equivalent (or ten years part-time equivalent) engaged in activities related to adults with dysphagia.

### 2.3. Procedure

#### 2.3.1. Recruitment

This study was approved by the Ethics Committee of Sud-Ouest et Outre Mer II (Comité de Protection des Personnes Sud-Ouest et Outre Mer II, France: 23.00117.000173). Delphi participants were recruited from the professional networks of the authors, professional organisations (e.g., the European Society for Swallowing Disorders [ESSD], the Union of European Phoniatricians [UEP] and the French Society for Deglutition and Dysphagia [SF2D]), and by asking recruited participants to identify other potential participants (snowballing). Identified potential participants were sent an invitation and information sheet about the background and purpose of the Delphi study. Participants who accepted the invitation received a link to the first online Delphi survey. All participants were reinvited for consecutive Delphi rounds regardless of whether they completed previous rounds, as survey data were processed anonymously.

#### 2.3.2. e-Delphi Surveys

A list of potential physiological impairments, risk factors and prognostic factors was constructed based on: (a) relevant international literature, and (b) group discussions between the authors. If required, definitions of the main concepts and references were listed. Potential factors were presented to participants across three Delphi rounds via an online survey platform (Limesurvey, University of Toulouse III–Paul Sabatier) over ten months (July 2022–April 2023).

Participants indicated consensus on the importance of a factor as a physiological impairment contributing to inefficient and/or unsafe swallowing, as a risk factor for the development of complications from oropharyngeal dysphagia, and/or as a prognostic factor for treatment outcome of oropharyngeal dysphagia and/or underlying etiology. Participants were also asked about the relevance of agreed factors as physiological impairments, risk factors and prognostic factors in clinical decision making, within the disease trajectory of oropharyngeal dysphagia. Participants responded to all questions on importance and relevance using a five-point scale. Participants who disagreed were asked to provide further details and comments in open text boxes. In addition, respondents were asked about the comprehensiveness of the lists of potential factors and to identify missing factors to fully capture all related constructs and aspects of the process of clinical decision making in oropharyngeal dysphagia. Finally, participants were requested to select factors that most contribute to the severity of oropharyngeal dysphagia from all included factors (i.e., physiological impairments, risk factors, and prognostic factors) as agreed on during the previous two Delphi rounds. Open-ended comment sections were available at the end of each Delphi survey. Between Delphi rounds, participants received summarised findings on participants’ demographics and percentage agreement on the importance of potential factors as physiological impairments, risk factors and prognostic factors. Supplementary File S1 provides samples of all three Delphi rounds with additional details on structure and content.

### 2.4. Analysis

Survey responses were analysed using the Statistical Package for the Social Sciences [15]. Before the first Delphi round, agreed consensus between participants was defined as at least 75% of respondents indicating ‘Strongly agree’ or ‘Agree’ on importance ratings (5-point scale: strongly disagree, disagree, neither agree nor disagree, agree, strongly agree), or ‘Extremely relevant’ or ‘Very relevant’ on relevance ratings (5-point scale: extremely relevant, very relevant, moderately relevant, slightly relevant, not at all relevant) [16,17]. Responses to open-ended questions on comprehensiveness were grouped into themes where potential new factors were identified based on the aggregated feedback. The final number of Delphi rounds was to be determined by the level of consensus following each round. The authors were blinded to participants’ identities when performing data analysis.

Exploratory Principal Component factor analyses were performed on relevance ratings of factors as physiological impairments, risk factors and prognostic factors in clinical decision making in the disease trajectory of oropharyngeal dysphagia. Furthermore, Mann–Whitney *U* tests were conducted to identify differences between professions (i.e., allied health professionals and medical specialists) in rating the relevance of factors in the clinical decision-making process. Differences between professions when ranking the ten factors most contributing to the severity of oropharyngeal dysphagia were compared using descriptive statistics.

## 3. Results

### 3.1. Delphi Participants

Potential candidates were identified through authors’ networks, professional organisations and snowballing. The numbers of participants who completed the three consecutive Delphi rounds were 75, 62 and 69, respectively. Table 1 presents the participants’ demographics. Across Delphi rounds, participants’ backgrounds varied slightly. Allied health professionals (49.3–60.1%) were mainly represented by speech–language pathologists (42.7–45.2%) in addition to occupational therapists and physiotherapists. About half of the medical specialists (39.9–50.7%) were otolaryngologists/phoniatricians (24.2–27.5%), whereas the remaining medical doctors represented a variety of specialties (e.g., radiologists, geriatricians, gastroenterologists and physiatrists). Most participants had completed a higher degree by research (62.3–62.9%) or Master’s degrees (29.0–36.7%), and showed wide variety in years of working with adults with dysphagia: 5–10 years (11.6–21.0%), 11–20 year (38.7–46.3%), and over 20 years of experience (41.9–44.0%). The majority of respondents were clinicians or clinical supervisors (71.0–76.0%) in addition to researchers (8.1–10.7%) and academics (12.0–14.5%), whose primary practice settings were mostly hospitals (66.7–75.8%), university/education (8.1–17.4%) and private practice (4.8–12.0%). Most frequently, participants worked with patients with non-degenerative neurological diseases (33.3–43.5%), oncology patients (23.2–24.2%) and geriatric populations (6.5–14.7%). Participants, of which over 80% originated from Europe, were spread across 31 countries and five continents. Further details can be found in Table 1.

### 3.2. Delphi Process

In addition to Supplementary File S1 providing an overview of the structure and content of all three Delphi rounds with listed example questions, a summary of the Delphi process is outlined in Table 2.

#### 3.2.1. Delphi Round I

The first Delphi round included: (1) a list of 22 items of potential physiological impairments, risk factors and prognostic factors (Part I), and (2) an additional 22 items of potential risk factors and prognostic factors only (Part II). Participants were asked to rate the importance of a factor as a physiological impairment contributing to inefficient and/or unsafe swallowing, a risk factor for the development of complications from oropharyngeal dysphagia, and/or a prognostic factor for treatment outcome or oropharyngeal dysphagia and/or underlying etiology. A total of 12 physiological impairments, 28 risk factors and 26 prognostic factors were identified achieving consensus ratings of 75% or higher. Participants were also asked about the comprehensibility of the presented items (Part III), resulting in an additional list of potential factors: 18 physiological impairments, 23 risk factors and 23 prognostic factors. Further details can be found in Table 3 (column ‘Importance’, Delphi Round I) presenting an overview of all potential factors and percentage agreement ratings resulting in the exclusion (<75% agreement) or inclusion (≥75% agreement) of relevant factors.

#### 3.2.2. Delphi Round II

The second Delphi round presented the carried-over new potential factors as suggested by the participants, using similar ‘*importance*’ rating scales. An additional total of nine physiological impairments, 15 risk factors and 13 prognostic factors were included based on participants’ agreement scores (Table 3 (column ‘Importance’, Delphi Round II). When asked about the comprehensiveness of all identified factors, no additional items were suggested by the participants.

#### 3.2.3. Delphi Round III

The final, third Delphi round focused on the *relevance* of agreed factors as physiological impairments (Part I), risk factors (Part II) and prognostic factors (Part III) in clinical decision making in the disease trajectory of oropharyngeal dysphagia. A total of 10 physiological impairments (10/21), 23 risk factors (23/43) and 21 prognostic factors (21/39) were identified as relevant factors in the clinical decision-making process (≥75% agreement). Table 3 (column ‘Relevance’) provides an overview of all relevance ratings.

Finally, participants were asked to select up to ten factors that most contribute to the severity of oropharyngeal dysphagia from all included fifty factors (i.e., physiological impairments, risk factors, and prognostic factors) as agreed on during the previous Delphi rounds (Part IV). Individual participants’ ranks ranged between 1–10, with 10 representing the most contributing factor to the severity of oropharyngeal dysphagia. The final ranking of factors was based on sum ranks. The five factors achieving the highest sum ranks were ‘Aspiration’, ‘Incomplete ejection or failure to eject aspirated materials from the airways’, ‘Weak or absent cough’, ‘Choking’ and ‘Sensory deficits in oropharynx’. When determining the frequency of factors listed as most contributing to the severity of oropharyngeal dysphagia, 40.6 to 68.1% of participants included the five factors with the highest sum ranks also in their individual top ten rankings. Further details on ranking results are provided in Table 3 (columns ‘Rank’ and ‘Frequency top 10’).

### 3.3. Statistical Analyses

#### 3.3.1. Exploratory Principal Component Factor Analyses

Three exploratory Principal Component factor analyses were performed for all physical impairments (*n* = 10), risk factors (*n* = 23) and prognostic factors (*n* = 21) that were considered relevant (≥75% agreement) in the process of clinical decision making for the disease trajectory of oropharyngeal dysphagia. The factor analysis including all relevant physical impairments revealed three components explaining 59.1% of the total variance (goodness-of-fit test, *p* < 0.001; Table 4 and Table 5); the factor analysis including all relevant risk factors identified six components explaining 74.2% of the total variance (goodness-of-fit test, *p* < 0.001; Table 6 and Table 7); and, the factor analysis including all relevant prognostic factors resulted in five components explaining 72.3% of the total variance (goodness-of-fit test, *p* < 0.001; Table 8 and Table 9). A factor analysis including all factors combined (i.e., physical impairments, risk factors and prognostic factors) was not performed due to poor sample-to-variable ratio (*N:p* ratio), referring to the number of participants (*n* = 69) compared to the total number of included factors (*n* = 53). Although recommendations differ in the literature, most rules of thumb suggest a minimum ratio of 3:1 [18].

Whereas some factors loaded on more than one component, obvious clustering of related factors was presented in all three exploratory factor analyses (Table 5, Table 7 and Table 9). For example, several factors related to contextual features (e.g., ‘No or limited assessment for oropharyngeal dysphagia’, ‘No or limited follow-up of people with oropharyngeal dysphagia’, ‘Lack of screening for oropharyngeal dysphagia’ and ‘Lack of skilled clinicians to manage and/or care for oropharyngeal dysphagia’) loaded on the same component (i.e., 1st and 5th component for analyses including risk and prognostic factors, respectively). Also, factors related to impaired airway protection (e.g., ‘Choking’, ‘Aspiration’ and ‘Incomplete ejection or failure to eject aspirated material from the airways’) loaded on the same component (3rd, 4th and 1st component, for analyses including physical impairments, risk factors and prognostic factors, respectively). Similar results were found for oral intake-related factors (i.e., ‘Malnutrition’ and ‘Insufficient or inadequate oral intake [e.g., caloric intake, consistency, nutritional supplements]’) loading on the 2nd and 4th component for risk and prognostic factors, respectively. As a third oral intake-related item, ‘Dehydration’ was not considered a prognostic factor; this item only loaded on the 2nd component for risk factors.

#### 3.3.2. Mann–Whitney *U* Tests and Ranking

Mann–Whitney *U* tests were conducted including the top five highest agreement ratings for the importance and relevance of Physical Impairment, Risk Factors and Prognostic Factors (Table 10). None of the thirty tests identified significant group differences between allied health professionals and medical specialists.

Similarly, group differences between professions were evaluated between the top ten rankings for factors that most contribute to the severity of oropharyngeal dysphagia (Table 11). Both allied health professionals and medical specialists included the following six factors in their top ten rankings (high to low-rank order): ‘Aspiration’, ‘Incomplete ejection or failure to eject aspirated materials from the airways’, ‘Weak or absent cough’, ‘Choking’, ‘Sensory deficits in oropharynx’ and ‘Poor oromotor functioning/skills (e.g., tongue, lip and velum)’. The remaining four top ten listed factors differed between professional groups but were still listed within the top 15 (medical specialists) or top 14 (allied health professionals) of the highest group rankings.

## 4. Discussion

### 4.1. Decision Making in Patients’ Disease Trajectory

Participant numbers ranged from 62 to 75 across three Delphi rounds. According to the COnsensus-based Standards for the selection of health Measurement INstruments (COSMIN) group, a minimum of 50 experts support very good methodological quality for quantitative studies such as this Delphi study [19]. Furthermore, the vast majority of participants (over 60%) had completed a higher degree by research or Master’s degree (around 30 to 35%), and most participants reported many years of experience working with adults with dysphagia, of whom over 40% reported more than 20 years of experience, indicating highly qualified and experienced Delphi participants.

After three Delphi rounds, a total of 29 factors were considered relevant in the clinical decision-making process (Table 3): 10 physiological impairments, 23 risk factors and 21 prognostic factors. Seven of these 29 factors were identified as relevant as a physiological impairment, risk factor and prognostic factor: ‘Aspiration’, ‘Incomplete ejection or failure to eject aspirated materials from the airways’, ‘Weak or absent cough’, ‘Choking’, ‘Sensory deficits in oropharynx’, ‘Poor oropharyngeal, hypopharyngeal and laryngeal motor functioning/skills’ and ‘Post-coma status and/or level of consciousness or alertness’. Ten factors were identified as both risk and prognostic factors, and one factor was identified as both a physical impairment and a risk factor. The remaining 11 factors were either considered a physical impairment (*n* = 2), a risk factor (*n* = 5) or a prognostic factor (*n* = 4).

When comparing the list of relevant factors with the four-topic approach by Jonsen et al. [11], a limited number of items referred to contextual features (e.g., ‘Caregiver dependence when eating [no or limited self-feeding skills]’, ‘Lack of social network/support [i.e., caregiver and family]’ and ‘Lack of skilled clinicians to manage and/or care for oropharyngeal dysphagia’), very few items referred to patient autonomy and preferences in treatment (e.g., ‘Nonadherence to treatment and medical recommendations’ and ‘Impaired cognitive functioning’), and no items referred to patient quality of life before and after treatment. As most factors refer to patient medical status, it could be argued that the extent to which a factor impacts clinical decision making in relation to disease trajectory (i.e., relevance ratings) or to oropharyngeal dysphagia (i.e., severity rankings), is predominantly determined by medical indications (diagnostic and therapeutic interventions, i.e., Jonsen’s fourth topic).

The concept of factor relevance, however, differed from the ranked contribution to the severity of oropharyngeal dysphagia. In relation to factors considered most contributory to dysphagia severity, some were considered highly relevant (≥80% agreement), but received relatively low severity rankings: e.g., ‘No or limited follow-up of people with oropharyngeal dysphagia’ (ranked 43), ‘Lack of social network/support (i.e., caregiver and family)’ (ranked 36) and ‘Lack of skilled clinicians to manage and/or care for oropharyngeal dysphagia’ (ranked 24) and ‘Unawareness of the swallowing disorder (i.e., anosognosia)’ (ranked 22).

When conducting exploratory Principal Component factor analyses for all physical impairments, risk factors and prognostic factors that were considered relevant, variables were grouped together, likely representing the same underlying construct or domain. Similar tendencies may be apparent from the ranking of variables contributing to the severity of dysphagia. For example, the top four ranked variables all referred to impaired airway protection (i.e., ‘Aspiration’, ‘Incomplete ejection or failure to eject aspirated materials from the airways’, ‘Weak or absent cough’ and ‘Choking’). Also, ‘Recurrent pulmonary infections’ (ranked 7) and ‘Poor respiratory status, pulmonary infection or pulmonary disease (e.g., COPD)’ (ranked 8) are related factors. Therefore, the ranked order of how factors contribute to dysphagia severity may need to be interpreted as representing different domains, instead of individual factors. Clinicians potentially compare and prioritise selected domains that represent a group of correlated individual factors when making decisions relating to patients’ disease trajectories. Similarly, differences were observed when comparing domain rankings between medical specialists and allied health professionals, for example, physicians generally ranked oral intake-related problems (i.e., ‘Insufficient or inadequate oral intake [e.g., caloric intake, consistency, nutritional supplements]’ and ‘Malnutrition’) significantly higher than allied health professionals.

### 4.2. Strengths and Limitations

The current Delphi study benefitted considerably from the incorporation of multiple opportunities for highly qualified and experienced participant feedback and discussion. Participants were encouraged to provide feedback throughout each survey using open text boxes for additional comments and to provide rationales for their ratings, or for feedback about the study in general. The authors used participant discussion and arguments to inform, for example, decisions about the inclusion of additional factors in progressive Delphi rounds. Further, participants were informed following each Delphi round about previous survey results, including decisions on excluded factors based on participant agreement ratings. However, while participants were considerably diverse in relation to geographical contexts (from 31 countries and five continents) and professional backgrounds, study outcomes remain highly dependent on the subjective perspectives of included participants.

Further, Exploratory Principal Component factor analyses were limited by poor sample-to-variable ratios. Therefore, analyses were conducted for each type of factor separately (i.e., physiological impairments, risk factors, and prognostic factors), but not for all factors combined. Combining the total number of relevant factors (*n* = 53) as determined by the current participants (*n* = 69) would have required higher recruitment numbers of at least triple the number of factors (*n* ≥ 159) [18].

## 5. Conclusions

This Delphi study reports on the first attempt to develop an international consensus on a clinical decision tree for the disease trajectory of oropharyngeal dysphagia in adults taking into account physiological impairments of swallowing, risk factors for the development of complications from oropharyngeal dysphagia, and prognostic factors for treatment outcome. A total of 10 physiological impairments, 23 risk factors and 21 prognostic factors were identified as relevant factors in the clinical decision-making process. Factors considered to contribute most to the severity of oropharyngeal dysphagia were ‘Aspiration’, ‘Incomplete ejection or failure to eject aspirated materials from the airways’, ‘Weak or absent cough’, ‘Choking’ and ‘Sensory deficits in oropharynx’. Future research is required to develop the current findings and confirm the domains based on relevant factors for clinical decision making, and those that contribute to the severity of oropharyngeal dysphagia, linking the current theoretical framework to clinical practice.

## Figures and Tables

**Table 1:** Participant demographics.

Round	Round One	Round Two	Round Three
Number of Participants	N = 75	N = 62	N = 69
Demographics	Frequency (%)	Frequency (%)	Frequency (%)
Continent of Residence			
Asia (Participants; Countries)	4 (5.3%; 4) (Hong Kong [*n* = 1], Lebanon [*n* = 1], Philippines [*n* = 1], United Arab Emirates [*n* = 1])	4 (6.5%; 4) (Hong Kong [*n* = 1], Japan [*n* = 1], Lebanon [*n* = 1], United Arab Emirates [*n* = 1])	6 (8.7%; 4) (Hong Kong [*n* = 1], Japan [*n* = 1], Turkey [*n* = 3], United Arab Emirates [*n* = 1])
Europe (Participants; Countries)	60 (80.0%; 20 ^a^)	50 (80.6%; 17 ^b^)	56 (81.2%; 19 ^c^)
North America (Participants; Countries)	3 (4.0%; 2) (Canada [*n* = 2], Unites States [*n* = 1])	2 (3.2%; 2) (Canada [*n* = 1], Unites States [*n* = 1])	2 (2.9%; 1) (Canada [*n* = 2])
Oceania (Participants; Countries)	3 (4.0%; 2) (Australia [*n* = 2], New Zealand [*n* = 1])	3 (4.8%; 2) (Australia [*n* = 2], New Zealand [*n* = 2])	1 (1.4%; 1) (Australia [*n* = 1])
South America (Participants; Countries)	5 (6.7%; 1) (Brazil [*n* = 5])	3 (4.8%; 1) (Brazil [*n* = 3])	4 (5.8%; 1) (Brazil [*n* = 4])
Highest qualification (related to work in the field of dysphagia)			
Bachelor	N.A.	5 (8.1%)	4 (5.8%)
Master	27 (36.0%)	18 (29.0%)	22 (36.7%)
PhD	47 (62.7%)	39 (62.9%)	43 (62.3%)
Profession			
Speech Language Pathologist	32 (42.7%)	28 (45.2%)	31 (44.9%)
Occupational Therapist	3 (4.0%)	4 (6.4%)	4 (5.8%)
Physiotherapist	2 (2.7%)	4 (6.4%)	3 (4.3%)
ENT/Phoniatrician	18 (24.0%)	15 (24.2%)	19 (27.5%)
Radiologist	6 (8.0%)	3 (4.8%)	4 (5.8%)
Other Medical Specialist	8 (10.7%)	5 (8.1%)	6 (8.7%)
Dual Allied Health Med Spec	6 (8.0%)	3 (4.8%)	N.A.
Primary role			
Clinician/Clinical supervisor	57 (76.0%)	46 (74.2%)	49 (71.0%)
Researcher	8 (10.7%)	5 (8.1%)	6 (8.7%)
Academic	9 (12.0%)	9 (14.5%)	9 (13.0%)
Teacher	N.A.	1 (1.6%)	1 (1.4%)
Not currently working	1 (1.3%)	N.A.	N.A.
Practice setting (Primary)			
Hospital	50 (66.7%)	47 (75.8%)	50 (72.5%)
Community	2 (2.7%)	3 (4.8%)	N.A.
Private Practice	9 (12.0%)	3 (4.8%)	5 (7.2%)
Residential Aged Care	1 (1.3%)	1 (1.6%)	2 (2.9%)
University/Education	12 (16.0%)	5 (8.1%)	12 (17.4%)
Student	1 (1.3%)	1 (1.6%)	N.A.
Other	N.A.	2 (3.2%) ^d^	N.A
Practice setting (secondary)			
No Secondary Sector	23 (30.7%)	13 (21.0%)	23 (33.3%)
Hospital	8 (10.7%)	3 (6.5%)	7 (10.1%)
Community	4 (5.3%)	3 (6.5%)	6 (8.7%)
Private Practice	5 (6.7%)	6 (9.7%)	6 (8.7%)
Residential Aged Care	3 (4.0%)	2 (3.2%)	1 (1.4%)
University/Education	26 (34.7%)	24 (38.7%)	20 (29%)
Student	6 (8.0%)	8 (12.9%)	6 (8.7%)
Patient populations (Primary)			
Non-degenerative Neurology	25 (33.3%)	26 (41.9%)	30 (43.5%)
Degenerative Neurology	8 (10.7%)	7 (11.3%)	2 (2.9%)
Oncology	18 (24.0%)	15 (24.2%)	16 (23.2%)
Gastroenterology	5 (6.7%)	1 (1.6%)	1 (1.4%)
Geriatrics	11 (14.7%)	4 (6.5%)	8 (11.6%)
Respiratory diseases	N.A.	1 (1.6%)	2 (2.9%)
Combined	7 (9.3%)	4 (6.5%)	6 (8.7%)
Other	N.A.	3 (4.8%)	N.A.
Patient populations (Secondary)			
No Secondary Population	5 (6.7%)	12 (19.4%)	5 (7.2%)
Non-degenerative Neurology	17 (22.7%)	11 (17.7%)	8 (11.6%)
Degenerative Neurology	22 (29.3%)	20 (32.3%)	22 (31.9%)
Oncology	8 (10.7%)	10 (16.1%)	8 (11.6%)
Gastroenterology	5 (6.7%)	1 (1.6%)	7 (10.1%)
Geriatrics	6 (8.0%)	2 (3.2%)	6 (8.7%)
Respiratory diseases	6 (8.0%)	5 (8.1%)	5 (7.2%)
Combined	4 (5.3%)	1 (1.6%)	4 (5.8%)
Other	1 (1.3%)	N.A.	N.A.
Years of experience			
5–10	13 (17.3%)	13 (21.0%)	8 (11.6%)
11–15	20 (26.7%)	14 (22.6%)	17 (24.6%)
16–20	9 (12.0%)	9 (14.5%)	15 (21.7%)
21–30	24 (32.0%)	19 (30.6%)	21 (30.4%)
>30	9 (12.0%)	7 (11.3%)	8 (11.6%)

Note. N.A. = Not Applicable. ^a^ Austria [*n* = 3], Belgium [*n* = 5], Croatia [*n* = 1], Denmark [*n* = 3], Estonia [*n* = 1], Finland [*n* = 2], France [*n* = 16], Germany [*n* = 3], Greece [*n* = 2], Italy [*n* = 3], Norway [*n* = 3], Portugal [*n* = 1], Slovakia [*n* = 2], Slovenia [*n* = 1], Spain [*n* = 1], Sweden [*n* = 3], Switzerland [*n* = 3], the Netherlands [*n* = 2], Ukraine [*n* = 1], United Kingdom [*n* = 4]. ^b^ Austria [*n* = 4], Belgium [*n* = 5], Croatia [*n* = 1], Denmark [*n* = 4], Finland [*n* = 1], France [*n* = 12], Germany [*n* = 2], Italy [*n* = 2], Norway [*n* = 2], Portugal [*n* = 1], Slovakia [*n* = 3], Slovenia [*n* = 2], Spain [*n* = 2], Sweden [*n* = 2], the Netherlands [*n* = 2], Ukraine [*n* = 1], United Kingdom [*n* = 4]. ^c^ Austria [*n* = 3], Belgium [*n* = 6], Croatia [*n* = 1], Denmark [*n* = 4], Estonia [*n* = 1], Finland [*n* = 2], France [*n* = 14], Germany [*n* = 3], Italy [*n* = 2], Norway [*n* = 2], Portugal [*n* = 2], Slovakia [*n* = 2], Slovenia [*n* = 2], Spain [*n* = 1], Sweden [*n* = 2], Switzerland [*n* = 2], the Netherlands [*n* = 2], Ukraine [*n* = 1], United Kingdom [*n* = 4]. ^d^ Currently not working (*n* = 1), Currently not working in dysphagia (*n* = 1).

**Table 2:** Overview of Delphi process.

Round (N_participants_)	Content	Results
Round I (N = 75)	**IMPORTANCE AND COMPREHENSIVENESS** **PART I List of potential factors: Physiological Impairments, Risk Factor and/or Prognostic Factor** (*n* = 22) **Questions ^a^ (5-point ordinal scale)** ▪ This factor is an important physiological impairment contributing to inefficient and/or unsafe swallowing. ▪ This factor is an important risk factor for the development of complications from oropharyngeal dysphagia. ▪ This factor is an important prognostic factor for treatment outcome or oropharyngeal dysphagia and/or underlying aetiology. **PART II List of potential factors: Risk Factor and/or Prognostic Factor** (*n* = 22) **Questions ^a,b^ (5-point ordinal scale)** ▪ This factor is an important risk factor for the development of complications from oropharyngeal dysphagia. ▪ This factor is an important prognostic factor for treatment outcome or oropharyngeal dysphagia and/or underlying aetiology. **PART III Comprehensiveness** Overview of all potential Physiological Impairments, Risk Factors and/or Prognostic Factors **(Open) Question** ▪ Focus on comprehensiveness; are any important factors missing?	**FACTORS Included: Agreement ^b^** ▪ Physiological Impairments (*n* = 12) ▪ Risk Factor (*n* = 28) ▪ Prognostic Factor (*n* = 26) **FACTORS Excluded: No agreement ^b^** ▪ Physiological Impairments (*n* = 10) ▪ Risk Factor (*n* = 16) ▪ Prognostic Factor (*n* = 18) **NEW potential FACTORS (Include Round II)** ▪ Physiological Impairments (*n* = 18) ▪ Risk Factor (*n* = 23) ▪ Prognostic Factor (*n* = 23)
Round II (N = 62)	**IMPORTANCE AND COMPREHENSIVENESS** **PART I List of new potential factors: Physiological Impairments, Risk Factors and/or Prognostic factors** from Round I, Part III (*n* = 18) **Questions** *as per Round I (Part I)* **PART II List of new Risk Factors and/or Prognostic factors** from Round I, Part III (*n* = 23) **Questions** *as per Round I (Part II)* **PART III Comprehensiveness** Overview of all potential Physiological Impairments, Risk Factors and/or Prognostic factors **(Open) Question** *as per Round I (Part III)*	**NEW FACTORS Included: Agreement ^b^** ▪ Physiological Impairments (*n* = 9) ▪ Risk Factor (*n* = 15) ▪ Prognostic factor (*n* = 13) **NEW FACTORS Excluded: No agreement ^b^** ▪ Physiological Impairments (*n* = 9) ▪ Risk Factor (*n* = 8) ▪ Prognostic factor (*n* = 10) **NEW potential FACTORS (Include Round III)** ▪ No new factors (*n* = 0)
Round III (N = 69)	**RELEVANCE AND RANK ORDER** **PART I List of included Physiological Impairments** (*n* = 21) **Question ^a,b^ (5-point ordinal scale)** ▪ Rate the relevance of the physiological impairment in clinical decision making in the disease trajectory of oropharyngeal dysphagia. **PART II List of included Risk Factors** (*n* = 43) **Question ^a,b^ (5-point ordinal scale)** ▪ Rate the relevance of the risk factor in clinical decision making in the disease trajectory of oropharyngeal dysphagia. **PART III List of included Prognostic Factors** (*n* = 39) **Question ^a,b^ (5-point ordinal scale)** ▪ Rate the relevance of the prognostic factor in clinical decision making in the disease trajectory of oropharyngeal dysphagia. **PART IV List of all included factors** (i.e., physiological impairments, risk factors, and prognostic factors) as agreed on during Round I and II (*n* = 50) **Question** ▪ Select and rank up to ten factors that most contribute to the severity of oropharyngeal dysphagia.	**FACTORS Included: Agreement ^b^** ▪ Physiological Impairments (*n* =10) ▪ Risk Factor (*n* = 23) ▪ Prognostic Factor (*n* = 20) **FACTORS Excluded: No agreement ^b^** ▪ Physiological Impairments (*n* = 11) ▪ Risk Factor (*n* = 21) ▪ Prognostic Factor (*n* = 18)

^a^ If ‘Disagree’ or ‘Strongly disagree’, changes can be suggested in comment boxes. ^b^ Consensus agreement is defined as ≥75% of participants rating ‘Strongly agree’ or ‘Agree’.

**Table 3:** Importance, Relevance and Ranking: Importance of factors as physiological impairment, risk factor, and/or prognostic factor; Relevance of factors in clinical decision making in the disease trajectory of oropharyngeal dysphagia; and, ranking of factors that most contribute to the severity of oropharyngeal dysphagia.

Item (Final Rank Order ^a^)	Importance ^b,c^ (% Agreement)				Relevance ^c,d^ (% Agreement)			Rank			Frequency top 10 ^e^	
	Physiological Impairment	Risk Factor	Prognostic Factor	Delphi Round	Physiological Impairment	Risk Factor	Prognostic Factor	Final Rank ^f^	Sum Rank ^g^	Mean Rank ^h^	Freq.	%
Aspiration	***84.0***	***94.6***	***86.6***	I	***91.3***	***92.8***	***85.5***	**1**	**390**	**5.7**	**46**	**66.7%**
Incomplete ejection or failure to eject aspirated materials from the airways	***93.3***	***98.6***	***89.4***	I	***98.6***	***92.8***	***85.5***	**2**	**357**	**5.2**	**47**	**68.1%**
Weak or absent cough	***94.7***	***100.0***	***89.4***	I	***85.7***	***91.3***	***87.0***	**3**	**344**	**5.0**	**44**	**63.8%**
Choking	***81.3***	***94.6***	***78.7***	I	***85.5***	***88.4***	***78.3***	**4**	**204**	**3.0**	**28**	**40.6%**
Sensory deficits in oropharynx	***96.0***	***97.3***	***88.0***	I	***78.3***	***79.7***	***76.8***	**5**	**187**	**2.7**	**36**	**52.2%**
Poor oromotor functioning/skills (e.g., tongue, lip and velum)	***97.3***	***81.3***	***81.4***	I	***86.9***	71.0	63.7	**6**	**182**	**2.6**	**26**	**37.7%**
Recurrent pulmonary infections	N.A.	***90.6***	***90.7***	I	N.A.	***88.4***	***84.1***	**7**	**151**	**2.2**	**30**	**43.5%**
Poor respiratory status, pulmonary infection or pulmonary disease (e.g., COPD)	***81.3***	***93.3***	***89.3***	I	73.9	***78.3***	***82.6***	**8**	**136**	**2.0**	**28**	**40.6%**
Progress and severity of underlying disease	***83.8***	***90.3***	***95.2***	II	72.1	***81.1***	***95.6***	**9**	**123**	**1.8**	**21**	**30.4%**
Presence of residue	***78.7***	***85.4***	70.7	I	71.0	***75.3***	N.A.	**10**	**116**	**1.7**	**23**	**33.3%**
Poor oropharyngeal, hypopharyngeal and laryngeal motor functioning/skills	***96.8***	***95.2***	***93.6***	II	***94.2***	***85.5***	***78.2***	11	113	1.6	24	34.8%
Insufficient or inadequate oral intake (e.g., caloric intake, consistency, nutritional supplements)	52.0	***85.4***	***81.3***	I	N.A.	***78.2***	***75.3***	12	107	1.6	19	27.5%
Malnutrition	64.0	***93.3***	***92.0***	I	N.A.	***78.2***	***79.7***	13	106	1.5	18	26.1%
Post-coma status and/or level of consciousness or alertness	***88.7***	***96.7***	***93.5***	II	***92.8***	***86.9***	***89.9***	14	105	1.5	15	21.7%
Frailty	N.A.	***93.3***	***86.6***	I	N.A.	66.6	***75.4***	15	81	1.2	17	24.6%
Poor oral hygiene	N.A.	***92.0***	***81.3***	I	N.A.	***75.4***	66.6	16	74	1.1	14	20.3%
Sarcopenia	***92.0***	***90.6***	***93.3***	I	58.1	69.5	71.0	17	72	1.0	13	18.8%
Impaired cognitive functioning	N.A.	***90.7***	***89.4***	I	N.A.	***81.1***	***84.0***	18	68	1.0	17	24.6%
Dehydration	N.A.	***90.7***	***85.4***	I	N.A.	***76.8***	73.9	19	65	0.9	13	18.8%
Anatomical/Physiological abnormalities	***87.1***	***83.9***	***82.3***	II	***75.3***	63.7	66.6	20	60	0.9	12	17.4%
Tracheostomy and/or ventilation support	***87.1***	***85.5***	***75.8***	II	***76.8***	***79.7***	71.0	21	57	0.8	12	17.4%
Unawareness of the swallowing disorder (i.e., anosognosia)	N.A.	***90.7***	***92.0***	I	N.A.	***82.6***	***81.2***	22	52	0.8	13	18.8%
Wet/gurgly voice	N.A.	***85.5***	***79.0***	II	N.A.	73.9	63.8	23	50	0.7	11	15.9%
Lack of skilled clinicians to manage and/or care for oropharyngeal dysphagia	56.5	***96.8***	***86.7***	II	N.A.	***85.5***	***90.9***	24	47	0.7	11	15.9%
Nonadherence to treatment and medical recommendations	N.A.	***81.3***	***82.7***	I	N.A.	72.4	***84.0***	25	42	0.6	11	15.9%
Respiratory difficulties	***92.0***	***75.1***	***88.7***	II	71.0	73.9	***78.3***	26	36	0.5	6	8.7%
No or limited assessment for oropharyngeal dysphagia	N.A.	***94.7***	***94.6***	I	N.A.	***84.0***	***75.4***	27	35	0.5	9	13.0%
Lack of screening for oropharyngeal dysphagia	N.A.	***89.3***	73.3	I	N.A.	***75.4***	N.A.	28	31	0.4	6	8.7%
Caregiver dependence when eating (no or limited self-feeding skills)	N.A.	***85.3***	***81.3***	I	N.A.	72.5	71.0	29	31	0.4	12	17.4%
Poor dental status	74.6	***78.7***	64.0	I	N.A.	47.8	N.A.	30	29	0.4	5	7.2%
Comorbidities (e.g., diabetes, COPD)	61.3	***80.0***	***76.0***	I	N.A.	59.4	71.0	31	27	0.4	7	10.1%
Drooling (i.e., sialorrhea)	***78.7***	66.7	54.7	I	43.4	N.A.	N.A.	32	26	0.4	5	7.2%
Chronicity of dysphagia	N.A.	***87.1***	***88.7***	II	N.A.	***75.3***	72.4	33	25	0.4	4	5.8%
Esophageal dysfunction or disease (e.g., reflux or GERD), or more general digestive problems	74.6	***81.4***	66.7	I	N.A.	53.6	N.A.	34	24	0.3	5	7.2%
Problems associated with head and body positioning (e.g, unable to support posture)	***86.6***	***94.7***	***84.0***	I	60.9	59.4	57.9	35	24	0.3	8	11.6%
Lack of social network/support (i.e., caregiver and family)	N.A.	64.0	***77.3***	I	N.A.	N.A.	***81.1***	36	23	0.3	7	10.1%
Xerostomia	***88.7***	74.2	69.4	II	43.4	N.A.	N.A.	37	19	0.3	4	5.8%
Poor mastication (not related to dentures)	***85.5***	***75.8***	56.5	II	58.0	49.3	N.A.	38	19	0.3	4	5.8%
Short attention span and/or poor memory	N.A.	58.0	***78.7***	I	N.A.	N.A.	69.6	39	18	0.3	5	7.2%
Lack of resources (e.g., adapted cutlery, special needs wheelchair, texture modified food)	N.A.	***82.7***	***85.3***	I	N.A.	66.7	71.0	40	15	0.2	6	8.7%
Apraxia	***77.4***	66.6	68.0	I	44.9	N.A.	N.A.	41	14	0.2	4	5.8%
Prolonged mealtime duration	62.9	74.2	***75.8***	II	N.A.	N.A.	50.7	42	13	0.2	6	8.7%
No or limited follow-up of people with oropharyngeal dysphagia	N.A.	***92.0***	***90.7***	I	N.A.	***82.6***	***81.2***	43	12	0.2	4	5.8%
Poor patient self-reported Functional Health Status (FHS)	53.3	***77.3***	***86.7***	I	N.A.	47.8	55.1	44	12	0.2	3	4.3%
Movement disorders	N.A.	***82.2***	***80.6***	II	N.A.	55.0	60.9	45	10	0.1	3	4.3%
Living environment (e.g., independent living, hospital, rehabilitation, nursing home)	N.A.	***83.9***	***83.9***	II	N.A.	56.5	68.1	46	9	0.1	2	2.9%
Delayed oral transit time	***85.5***	72.5	71.0	II	47.8	N.A.	N.A.	47	8	0.1	2	2.9%
Bedridden	61.3	***82.2***	72.5	II	N.A.	65.2	N.A.	48	4	0.1	2	2.9%
Poor self-feeding skills	71.0	***83.9***	***80.6***	II	N.A.	69.6	62.3	49	3	0.0	1	1.4%
Infections of the oral cavity, naso, oro and/or hypopharynx	70.9	***77.4***	66.2	II	N.A.	63.6	N.A.	50	2	0.0	1	1.4%
Advanced age	N.A.	74.7	64.7	I	N.A.	N.A.	N.A.	N.A.	N.A.	N.A.	N.A.	N.A.
Aphasia and/or poor communication skills	43.6	41.9	53.2	II	N.A.	N.A.	N.A.	N.A.	N.A.	N.A.	N.A.	N.A.
Behavioural/psychological eating disorders (e.g., anorexia, picky eaters, binge eating)	N.A.	58.0	50.7	I	N.A.	N.A.	N.A.	N.A.	N.A.	N.A.	N.A.	N.A.
Dependence in activities of daily living (ADL)	N.A.	69.3	70.6	I	N.A.	N.A.	N.A.	N.A.	N.A.	N.A.	N.A.	N.A.
Depression	N.A.	45.3	66.7	I	N.A.	N.A.	N.A.	N.A.	N.A.	N.A.	N.A.	N.A.
Dysarthria	59.6	50.0	54.8	II	N.A.	N.A.	N.A.	N.A.	N.A.	N.A.	N.A.	N.A.
Dysphonia	42.0	48.4	43.5	II	N.A.	N.A.	N.A.	N.A.	N.A.	N.A.	N.A.	N.A.
Geographical isolation (no or limited access to support)	N.A.	58.7	66.7	I	N.A.	N.A.	N.A.	N.A.	N.A.	N.A.	N.A.	N.A.
Inappropriate impulsive behaviour	53.3	68.0	62.7	I	N.A.	N.A.	N.A.	N.A.	N.A.	N.A.	N.A.	N.A.
Lack of inhibitory control on food intake	57.4	73.3	64.0	I	N.A.	N.A.	N.A.	N.A.	N.A.	N.A.	N.A.	N.A.
Loss of appetite	30.6	66.7	57.3	I	N.A.	N.A.	N.A.	N.A.	N.A.	N.A.	N.A.	N.A.
No or limited supervision during mealtimes	N.A.	65.3	62.6	I	N.A.	N.A.	N.A.	N.A.	N.A.	N.A.	N.A.	N.A.
Orofacial, laryngeal and/or pharyngeal pain	74.2	58.1	66.2	II	N.A.	N.A.	N.A.	N.A.	N.A.	N.A.	N.A.	N.A.
Penetration	68.0	70.7	61.4	I	N.A.	N.A.	N.A.	N.A.	N.A.	N.A.	N.A.	N.A.
Polypharmacy	N.A.	72.6	67.7	II	N.A.	N.A.	N.A.	N.A.	N.A.	N.A.	N.A.	N.A.
Social isolation	N.A.	60.0	70.6	I	N.A.	N.A.	N.A.	N.A.	N.A.	N.A.	N.A.	N.A.
Tube feeding	N.A.	66.7	69.4	I	N.A.	N.A.	N.A.	N.A.	N.A.	N.A.	N.A.	N.A.

Note. N.A. (grey background) = Not Applicable. ^a^ Items ordered as per final ranks. Items not considered important physiological impairments, risk factors or prognostic factors are listed in alphabetical order at the end of the table. ^b^ Importance of factors as physiological impairment, risk factor, and/or prognostic factor. ^c^ Data in bold italic: % Agreement ≥ 75% (Agree or Strongly Agree). ^d^ Relevance of factors (i.e., physiological impairment, risk factor, and/or prognostic factor) in clinical decision making in the disease trajectory of oropharyngeal dysphagia. ^e^ Frequency of items being included in the top ten factors that most contribute to the severity of oropharyngeal dysphagia (N_Total_ = 69). ^f^ Rank order based on sum of all ranks. ^g^ Sum of all participants’ ranks (N_Total_ = 69); individual participants’ ranks ranging between 1–10 (with 10 representing the most contributing factor to the severity of oropharyngeal dysphagia). ^h^ Mean of all participants’ ranks (N_Total_ = 69).

**Table 4:** Exploratory Principal Component factor analyses for Physical Impairments relevant in the process of clinical decision making for the disease trajectory of oropharyngeal dysphagia: Eigenvalues, Percentages of variance and Cumulative percentages for components for 10 physical impairment items.

Component	Eigenvalue	% of Variance	Cumulative %
1	2.938	29.384	29.384
2	1.637	16.370	45.754
3	1.334	13.342	59.096

**Table 5:** Exploratory Principal Component factor analyses for Physical Impairments: Factor loadings and Communalities for Varimax rotated 3-Factor solution for 10 items (*n* = 69).

Physical Impairment	Component			Communalities
Item	1	2	3	
Sensory deficits in oropharynx	**0.804**	0.115	0.267	0.731
Poor oromotor functioning/skills (e.g., tongue, lip and velum)	**0.750**	−0.283	0.155	0.666
Poor oropharyngeal and laryngeal motor functioning/skills	**0.686**	0.360	−0.131	0.618
Anatomical/Physiological abnormalities	**0.640**	0.399	−0.158	0.594
Post-coma status and/or level of consciousness or alertness	0.244	**0.718**	0.168	0.604
Tracheostomy and/or ventilation support	0.185	**0.647**	0.162	0.479
Weak or absent cough	−0.057	**0.528**	−0.006	0.282
Choking	0.069	−0.235	**0.777**	0.664
Incomplete ejection or failure to eject aspirated materials from the airways	−0.130	0.347	**0.724**	0.662
Aspiration	0.208	0.308	**0.687**	0.610

Factor loadings over 0.40 appear in bold. Highest factor loading per item appears against a dark grey background.

**Table 6:** Exploratory Principal Component factor analyses for Risk Factors relevant in the process of clinical decision making for the disease trajectory of oropharyngeal dysphagia: Eigenvalues, Percentages of variance and Cumulative percentages for components for 23 risk factor items.

Component	Eigenvalue	% of Variance	Cumulative %
1	8.879	38.604	38.604
2	2.273	9.884	48.488
3	2.054	8.929	57.416
4	1.486	6.461	63.878
5	1.300	5.653	69.531
6	1.081	4.702	74.233

**Table 7:** Exploratory Principal Component factor analyses for Risk Factors: Factor loadings and Communalities for Varimax rotated 6-Factor solution for 23 items (*n* = 69).

Risk Factor	Component						Communalities
Item	1	2	3	4	5	6	
No or limited assessment for oropharyngeal dysphagia	**0.905**	0.082	0.153	0.077	0.177	0.043	0.888
No or limited follow-up of people with oropharyngeal dysphagia	**0.877**	0.065	0.164	0.067	0.222	0.060	0.859
Lack of screening for oropharyngeal dysphagia	**0.819**	0.121	0.120	0.209	−0.209	0.167	0.816
Lack of skilled clinicians to manage and/or care for oropharyngeal dysphagia	**0.698**	0.128	0.216	0.086	0.321	0.294	0.747
Progress and severity of underlying disease	**0.584**	0.168	0.272	0.060	**0.492**	0.211	0.734
Malnutrition	0.148	**0.887**	0.159	0.028	0.116	0.049	0.850
Dehydration	0.130	**0.766**	0.157	0.194	0.132	0.113	0.697
Insufficient or inadequate oral intake (e.g., caloric intake, consistency, nutritional supplements)	−0.007	**0.838**	0.131	−0.012	0.091	0.211	0.772
Poor oral hygiene	0.252	**0.497**	−0.175	0.196	**0.406**	−0.014	0.544
Poor oropharyngeal, hypopharyngeal and laryngeal motor functioning/skills	0.258	0.134	**0.764**	0.242	0.032	.072	0.732
Chronicity of dysphagia	0.135	−0.107	**0.682**	0.017	0.278	.316	0.672
Sensory deficits in oropharynx	0.326	0.258	**0.657**	0.238	−0.114	0.195	0.713
Presence of residue	0.075	0.316	**0.514**	0.367	0.150	0.038	0.529
Post-coma status and/or level of consciousness or alertness	0.259	0.286	**0.467**	0.275	**0.417**	0.187	0.651
Incomplete ejection or failure to eject aspirated materials from the airways	0.051	0.109	0.260	**0.841**	0.207	0.047	0.834
Choking	0.250	0.111	−0.006	**0.820**	−0.141	0.229	0.820
Aspiration	0.083	0.095	0.372	**0.727**	0.316	0.089	0.790
Tracheostomy and/or ventilation support	0.287	0.209	0.283	0.108	**0.747**	0.088	0.784
Weak or absent cough	0.071	−0.092	0.227	0.485	**0.509**	0.355	0.685
Poor respiratory status, pulmonary infection or pulmonary disease (e.g., COPD)	0.062	**0.488**	−0.085	0.112	**0.704**	0.172	0.788
Recurrent pulmonary infections	0.118	−0.006	0.081	0.284	0.244	**0.749**	0.722
Impaired cognitive functioning	0.136	0.375	0.384	0.004	−0.012	**0.690**	0.783
Unawareness of the swallowing disorder (i.e., anosognosia)	0.285	0.305	0.154	0.123	0.103	**0.664**	0.665

Factor loadings over 0.40 appear in bold. Highest factor loading per item appears against a dark grey background and second highest factor loading against a light grey background (if > 0.40).

**Table 8:** Exploratory Principal Component factor analyses for Prognostic Factors relevant in the process of clinical decision making for the disease trajectory of oropharyngeal dysphagia: Eigenvalues, Percentages of variance and Cumulative percentages for components for 21 prognostic factor items.

Component	Eigenvalue	% of Variance	Cumulative %
1	8.423	40.108	40.108
2	2.345	11.166	51.275
3	1.980	9.427	60.702
4	1.337	6.365	67.067
5	1.094	5.207	72.275

**Table 9:** Exploratory Principal Component factor analyses for Prognostic Factors: Factor loadings and Communalities for Varimax rotated 5-Factor solution for 21 items (*n* = 69).

Prognostic Factor	Component					Communalities
Item	1	2	3	4	5	
Aspiration	**0.886**	0.144	0.167	0.072	0.006	0.839
Incomplete ejection or failure to eject aspirated materials from the airways	**0.864**	0.157	0.082	0.210	0.079	0.828
Weak or absent cough	**0.826**	−0.002	−0.012	0.076	0.051	0.691
Poor oropharyngeal, hypopharyngeal and laryngeal motor functioning/skills	**0.776**	0.294	0.262	0.072	0.027	0.763
Choking	**0.759**	0.131	0.216	0.002	0.081	0.647
Sensory deficits in oropharynx	**0.737**	0.308	0.144	0.264	0.036	0.730
Post-coma status and/or level of consciousness or alertness	**0.553**	0.343	−0.053	0.317	0.373	0.666
Impaired cognitive functioning	0.182	**0.806**	−0.050	0.067	0.219	0.738
Unawareness of the swallowing disorder (i.e., anosognosia)	0.095	**0.789**	0.014	0.272	0.083	0.713
Nonadherence to treatment and medical recommendations	0.343	**0.778**	0.208	0.052	−0.024	0.770
Lack of social network/support (i.e., caregiver and family)	0.120	**0.720**	0.218	0.087	0.102	0.598
Insufficient or inadequate oral intake (e.g., caloric intake, consistency, nutritional supplements)	0.257	0.119	**0.870**	0.040	0.073	0.843
Malnutrition	0.082	0.150	**0.836**	0.203	0.087	0.777
Poor respiratory status, pulmonary infection or pulmonary disease (e.g., COPD)	0.139	−0.052	**0.552**	**0.424**	**0.449**	0.708
Recurrent pulmonary infections	**0.505**	0.094	**0.513**	0.251	0.147	0.612
No or limited assessment for oropharyngeal dysphagia	0.151	0.136	0.264	**0.851**	0.021	0.835
No or limited follow-up of people with oropharyngeal dysphagia	0.228	0.265	0.124	**0.776**	0.078	0.746
Lack of skilled clinicians to manage and/or care for oropharyngeal dysphagia	0.180	0.029	0.038	0.374	−0.594	0.526
Respiratory difficulties	0.239	0.130	**0.406**	0.320	**0.674**	0.796
Progress and severity of underlying disease	0.281	**0.426**	0.083	0.065	**0.658**	0.704
Frailty	0.073	0.280	0.297	**0.405**	**0.557**	0.646

Factor loadings over 0.40 appear in bold. Highest factor loading per item appear against a dark grey background and second highest factor loading against a light grey background (if > 0.40).

**Table 10:** Group differences between allied health professionals and medical specialists for item agreement ratings (top five): Mann–Whitney *U* test.

	Factor	Item (Top Five)	% Agreement (All Participant)	AH	MS	U	*p*-Value (Two-Tailed)
				MN Rank (*n*)	MN Rank (*n*)		
**Importance ^a^**	**Physical Impairment**	Poor oromotor functioning/skills (e.g., tongue, lip and velum)	97.3	36.78 (38)	39.26 (37)	749.5	0.570
		Poor oropharyngeal, hypopharyngeal and laryngeal motor functioning/skills	96.8	30.68 (36)	32.36 (26)	497.5	0.621
		Sensory deficits in oropharynx	96.0	38.95 (38)	37.03 (37)	667.0	0.660
		Weak or absent cough	94.7	35.32 (38)	40.76 (37)	805.0	0.174
		Incomplete ejection or failure to eject aspirated materials from the airways	93.3	36.61 (38)	39.43 (37)	756.0	0.487
	**Risk Factor**	Weak or absent cough	100	37.57 (38)	38.45 (37)	719.5	0.756
		Incomplete ejection or failure to eject aspirated materials from the airways	98.6	37.21 (38)	38.81 (37)	733.0	0.638
		Sensory deficits in oropharynx	97.3	40.09 (38)	35.85 (37)	623.5	0.332
		Lack of skilled clinicians to manage and/or care for oropharyngeal dysphagia	96.8	33.24 (36)	29.10 (26)	405.5	0.310
		Post-coma status and/or level of consciousness or alertness	96.7	30.11 (36)	33.42 (26)	518.0	0.382
	**Prognostic Factor**	Progress and severity of underlying disease	95.2	32.89 (36)	29.58 (26)	418.0	0.368
		No or limited assessment for oropharyngeal dysphagia	94.6	38.71 (38)	37.27 (37)	676.0	0.747
		Poor oropharyngeal, hypopharyngeal and laryngeal motor functioning/skills	93.6	31.67 (36)	31.27 (26)	462.0	0.921
		Post-coma status and/or level of consciousness or alertness	93.5	30.63 (36)	32.71 (26)	499.5	0.610
		Sarcopenia	93.3	39.89 (38)	36.05 (37)	631.0	0.392
**Relevance ^b^**	**Physical Impairment**	Incomplete ejection or failure to eject aspirated materials from the airways	98.6	34.45 (38)	35.45 (31)	572.0	0.784
		Poor oropharyngeal, hypopharyngeal and laryngeal motor functioning/skills	94.2	35.06 (38)	34.95 (31)	591.0	0.978
		Post-coma status and/or level of consciousness or alertness	92.8	32.19 (38)	37.29 (31)	502.0	0.240
		Aspiration	91.3	36.24 (38)	33.99 (31)	627.5	0.576
		Poor oromotor functioning/skills (e.g., tongue, lip and velum)	86.9	32.71 (38)	36.87 (31)	518.0	0.345
	**Risk Factor**	Incomplete ejection or failure to eject aspirated materials from the airways	92.8	34.97 (38)	35.03 (31)	590.0	0.988
		Aspiration	92.8	35.94 (38)	34.24 (31)	618.0	0.679
		Weak or absent cough	91.3	31.66 (38)	37.72 (31)	485.5	0.145
		Recurrent pulmonary infections	88.4	32.58 (38)	36.97 (31)	514.0	0.315
		Choking	88.4	35.48 (38)	34.61 (31)	604.0	0.841
	**Prognostic Factor**	Progress and severity of underlying disease	95.6	33.48 (38)	36.24 (31)	542.0	0.519
		Post-coma status and/or level of consciousness or alertness	89.9	35.02 (38)	34.99 (31)	589.5	0.995
		Weak or absent cough	87.0	37.26 (38)	33.16 (31)	659.0	0.353
		Incomplete ejection or failure to eject aspirated materials from the airways	85.5	36.94 (38)	33.42 (31)	649.0	0.424
		Aspiration	85.5	38.21 (38)	32.38 (31)	688.5	0.190

^a^ Importance of factors as physiological impairment, risk factor, and/or prognostic factor. ^b^ Relevance of factors (i.e., physiological impairment, risk factor, and/or prognostic factor) in clinical decision making in the disease trajectory of oropharyngeal dysphagia.

**Table 11:** Ranking of factors that most contribute to the severity of oropharyngeal dysphagia: differences in ranking between allied health professionals and medical specialists.

Item	All Participants (*n* = 69)			Allied Health (*n* = 38)			Medical Specialists (*n* = 31)		
	Final Rank	Sum Rank	MN Rank (Sum Rank/*n*)	Final Rank	Sum Rank	MN Rank (Sum Rank/*n*)	Final Rank	Sum Rank	MN Rank (Sum Rank/*n*)
Aspiration	1	390	5.65	1	211	5.55	1	179	5.77
Incomplete ejection or failure to eject aspirated materials from the airways	2	357	5.17	2	185	4.87	2	172	5.55
Weak or absent cough	3	344	4.98	3	180	4.74	3	164	5.29
Choking	4	204	2.96	6	95	2.50	4	109	3.52
Sensory deficits in oropharynx	5	187	2.71	4	132	3.47	10	55	1.77
Poor oromotor functioning/skills (e.g., tongue, lip and velum)	6	182	2.64	7	93	2.45	6	89	2.87
Recurrent pulmonary infections	7	151	2.19	14	47	1.24	5	104	3.36
Poor respiratory status, pulmonary infection or pulmonary disease (e.g., COPD)	8	136	1.97	5	102	2.68	15	34	1.10
Progress and severity of underlying disease	9	123	1.78	8	81	2.13	12	42	1.36
Presence of residue	10	116	1.68	11	57	1.50	9	59	1.90
Poor oropharyngeal, hypopharyngeal and laryngeal motor functioning/skills	11	113	1.64	10	64	1.68	11	49	1.58
Insufficient or inadequate oral intake (e.g., caloric intake, consistency, nutritional supplements)	12	107	1.55	18	41	1.08	7	66	2.13
Malnutrition	13	106	1.54	16	46	1.21	8	60	1.94
Post-coma status and/or level of consciousness or alertness	14	105	1.52	9	67	1.76	13	38	1.23
Frailty	15	81	1.17	15	46	1.21	14	35	1.13

Note. Data against a grey background refer to items in the group’s top ten (i.e., all participants, allied health professionals and/or medical specialists).

## Data Availability

Data is available on request due to ethical restrictions.

## Supplementary Materials

The following supporting information can be downloaded at: https://www.mdpi.com/article/10.3390/jcm12206572/s1, Supplementary File S1: Structure and content of Delphi rounds.
