# Supplementary material for: Expert Consensus on Clinical Decision Making in the Disease Trajectory of Oropharyngeal Dysphagia in Adults: An International Delphi Study
Source: J Clin Med. 2023 Oct 17;12(20):6572. doi: 10.3390/jcm12206572 (PMC10607151; doi:10.3390/jcm12206572)
Supplement: Supplementary file 1 [file jcm-12-06572-s001.zip › jcm-2626396-supplementary.pdf]

Expert Consensus on Clinical Decision-Making in the Disease Trajectory of Oropharyngeal Dysphagia:

an international Delphi Study

Supplementary file S1. Structure and content of Delphi rounds.

ROUND I

- *Eligibility criteria* (fluency in English, working with adults with dysphagia);
- Questions on *Demographics* (profession, sector, qualification, primary role, patient populations, qualification, years of experience, country);
- Definitions of key *terminology* in clinical decision-making in the disease trajectory of oropharyngeal dysphagia.

1) **Physiological Impairment** *of swallowing (or swallowing disability) resulting in inefficient and/or unsafe swallowing*. Swallowing efficiency refers to the ability to transfer a bolus, secretions and/or any other material from the oral cavity, nasal cavity and paranasal sinuses, tracheobronchial tree or esophagus to the stomach without post-swallow residue, whereas swallowing safety refers to the ability to transfer to the stomach without penetration and/or aspiration into the lower airways.
2) **Risk Factors** *for the development of complications from oropharyngeal dysphagia (e.g., pulmonary complications or aspiration pneumonia, reduced nutrition or oral intake, and/or health-related quality-of-life or depression) referring to (1) patient characteristics in relation to patient's health status or comorbidities, and/or (2) health consequences due to physiological impairment of swallowing* (as defined under 1.).
For example, health consequences due to tracheal residue (physiological impairment: see 1.) may lead to the development of aspiration pneumonia (complication) in presence of pulmonary disease, e.g., COPD (risk factor: see 2.).
3) **Prognostic Factors** *for treatment outcome of oropharyngeal dysphagia and/or treatment of underlying etiology.* Prognostic factors may impact the success of any treatment for oropharyngeal dysphagia and/or treatment of underlying etiology.

PART I

- List of potential factors (i.e., *physiological impairments, risk factors, and prognostic factors*);
- *Questions on the importance* of each factor that may be considered as physiological impairment, risk factor, and/or prognostic factor (using 5-point ordinal scales and open text boxes for additional comments).

EXAMPLE Question

Weak or absent cough *

Question. This factor is an important physiological impairment contributing to inefficient and/or unsafe swallowing:

- ☐ Strongly disagree
- ☐ Disagree
- ☐ Neither agree nor disagree
- ☐ Agree
- ☐ Strongly agree

[Open text box if 'Strongly disagree' or 'Disagree']
Please indicate why you think this item is not an important physiological impairment contributing to inefficient and/or unsafe swallowing.

Question. This factor is an important risk factor for the development of complications from oropharyngeal dysphagia:

- Strongly disagree
- Disagree
- Neither agree nor disagree
- Agree
- Strongly agree

[Open text box if 'Strongly disagree' or 'Disagree']
Please indicate why you think this item is not an important risk factor for the development of complications from oropharyngeal dysphagia.

Question. This factor is an important prognostic factor for treatment outcome or oropharyngeal dysphagia and/or underlying etiology:

- Strongly disagree
- Disagree
- Neither agree nor disagree
- Agree
- Strongly agree

[Open text box if 'Strongly disagree' or 'Disagree']
Please indicate why you think this item is not an important prognostic factor for treatment outcome or oropharyngeal dysphagia and/or underlying etiology.

* Cough: Airway protective behaviour or reflex which responds to material entering the airway by producing high velocity airflows creating shearing forces in larger airways and squeezing actions in smaller airways to remove mucus and foreign matters (Ross et al., 1955; Fontana & Lavorini, 2006; Widdicombe & Chung, 2007)

PART II

- List of potential factors (i.e., *risk factors and prognostic factors*), other than physiological impairments;
- *Questions on the importance* of each factor that may be considered as a risk factor, and/or prognostic factor (using 5-point ordinal scales and open text boxes for additional comments).

EXAMPLE Factors
Advanced age
Frailty
Dependence in activities of daily living (ADL)

EXAMPLE Questions *(Risk factor and prognostic factor)*
As per PART I.

PART III

- Overview of all potential factors (i.e., physiological impairments, risk factors, and prognostic factors); Question (three open text boxes) to identify any important physiological impairments, risk factors, and prognostic factors, that had not been listed (*comprehensiveness*).

ROUND II

- *Summary of Round I results*;
- *Eligibility criteria* (fluency in English, working with adults with dysphagia);
- Questions on *Demographics* (profession, sector, qualification, primary role, patient populations, qualification, years of experience, country).

PART I

- Additional factors identified by participants during Round I (Part III).
  Questions on the importance of each factor that may be considered as a *physiological impairment, risk factor, and/or prognostic factor* as per Round I (Part I).

PART II

- Additional factors identified by participants during Round I (Part III).
  Questions on the importance of each factor that may be considered as a *risk factor, and/or prognostic factor* as per Round I (Part II).

PART III

- Overview of all potential factors (i.e., physiological impairments, risk factors, and prognostic factors); Question (three open text boxes) to identify any important physiological impairments, risk factors, and prognostic factors, that had not been listed (*comprehensiveness*).

- Open text box to add any other comments.

ROUND III

- *Summary of Round I results*;
- *Eligibility criteria* (fluency in English, working with adults with dysphagia);
- Questions on *Demographics* (profession, sector, qualification, primary role, patient populations, qualification, years of experience, country).

PART I

- List of included *physiological impairments* as agreed on during Round I and II.
  Question on the relevance of each factor in the clinical decision-making in the disease trajectory of oropharyngeal dysphagia using 5-point ordinal scales and open text boxes for additional comments.
- Open text box to add any other comments.

PART II

- List of included *risk factors* as agreed on during Round I and II.
  Question on the relevance of each factor in the clinical decision-making in the disease trajectory of oropharyngeal dysphagia using 5-point ordinal scales and open text boxes for additional comments.
- Open text box to add any other comments.

PART III

- List of included *prognostic factors* as agreed on during Round I and II.
  Question on the relevance of each factor in the clinical decision-making in the disease trajectory of oropharyngeal dysphagia using 5-point ordinal scales and open text boxes for additional comments.
- Open text box to add any other comments.

EXAMPLE Question

Question. Rate the relevance of the following risk factor in clinical decision-making in the disease trajectory of oropharyngeal dysphagia.

Progress and severity of underlying disease:

- ☐ Extremely relevant
- ☐ Very relevant
- ☐ Moderately relevant
- ☐ Slightly relevant
- ☐ Not at all relevant

NB. Relevance refers to the degree to which a risk factor contributes to clinical decision-making in the disease trajectory of oropharyngeal dysphagia.

PART IV

- Overview of all included factors (i.e., physiological impairments, risk factors, and prognostic factors) as agreed on during Round I and II.
  Question to select and rank up to ten factors that most contribute to the severity of oropharyngeal dysphagia.

- Open text box to add any other comments.
